# Lateral medullary vascular compression manifesting as paroxysmal hypertension

**DOI:** 10.1007/s00701-024-06032-y

**Published:** 2024-03-15

**Authors:** L. Giammattei, G. Wuerzner, K. Theiler, P. Vollenweider, V. Dunet, M. Al Barajraji, J. W. Squair, J. Bloch, R. T. Daniel

**Affiliations:** 1 Department of Neurosurgery, Lausanne University Hospital, Lausanne and University of Lausanne, Lausanne, Switzerland; 2 Service of Nephrology and Hypertension, Lausanne University Hospital and University of Lausanne, Lausanne, Switzerland; 3 Department of Medicine, Internal Medicine, Lausanne University Hospital and University of Lausanne, Lausanne, Switzerland; 4 Department of Medical Radiology, Service of Diagnostic and Interventional Radiology, Neuroradiology Unit, Lausanne University Hospital and University of Lausanne, Lausanne, Switzerland; 5 Defitech Center for Interventional Neurotherapies (NeuroRestore), CHUV/UNIL/EPFL, 1005 Lausanne, Switzerland; 6 Department of Clinical Neuroscience, Lausanne University Hospitzal (CHUV) and University of Lausanne (UNIL), 1005 Lausanne, Switzerland

**Keywords:** Far lateral approach, Neurogenic hypertension, Microvascular decompression, Ventrolateral medullary decompression, Symptomatic paroxysmal hypertension, Vago-glossopharyngeal neuralgia

## Abstract

Neurovascular compression of the rostral ventrolateral medulla (RVLM) has been described as a possible cause of refractory essential hypertension. We present the case of a patient affected by episodes of severe paroxysmal hypertension, some episodes associated with vago-glossopharyngeal neuralgia. Classical secondary forms of hypertension were excluded. Imaging revealed a neurovascular conflict between the posterior inferior cerebellar artery (PICA) and the ventrolateral medulla at the level of the root entry zone of the ninth and tenth cranial nerves (CN IX-X REZ). A MVD of a conflict between the PICA and the RVLM and adjacent CN IX-X REZ was performed, resulting in reduction of the frequency and severity of the episodes. Brain MRI should be performed in cases of paroxysmal hypertension. MVD can be considered in selected patients.

**Supplementary Information:**

The online version contains supplementary material available at 10.1007/s00701-024-06032-y.

## Introduction

In 1979, Jannetta reported a possible connection between essential hypertension and neurovascular compression of the rostral ventrolateral medulla (RVLM) at the level of root entry zone of the 9th and 10th cranial nerves (CN IX-X REZ) [10]. The RVLM regulates sympathetic activity through descending sympatho-excitatory axonal projections and is involved in tonic and dynamic cardiovascular reflexes [19, 21]. Consequently, vascular compression of this region could lead to physical perturbation of C1 neurons of the RVLM, or deafferentation of the nucleus tractus solitarius, both of which are involved in the natural baroreflex arc [11]. This pathophysiological background, characterized by chronic or transient increases in sympathetic activity, led to microvascular decompression (MVD) being nominated as a possible treatment of refractory neurogenic hypertension. Despite many uncertainties and only small cohorts undergoing this approach, MVD remains an option among patients with proven refractory hypertension that experience life-threatening uncontrolled blood pressure changes despite multiple anti-hypertensive agents.

A particular subset of these patients with neurogenic hypertension are diagnosed with paroxysmal hypertension, characterized by markedly symptomatic and abrupt episodes of blood pressure elevation along with headache, nausea, chest discomfort, dizziness, and other neurological symptoms. These patients do not usually describe a triggering emotional distress and psychiatric consults suggestive of a panic disorder [14]. Here, we describe a case of a patient with paroxysmal hypertension and occasionally concomitant glossopharyngeal neuralgia where the brain MRI showed a neurovascular conflict between the left posterior inferior cerebellar artery (PICA) and the RVLM and adjacent CN IX-X REZ. This conflict was successfully treated though MVD.

## Case description

A 78-year-old patient had a past medical history of atrial fibrillation, gastritis, monoclonal gammopathy, hip replacement, and pulmonary-vein isolation for atrial fibrillation. Three months after the cardiac procedure and 2 years before his presentation to us, he started developing episodes of paroxysmal hypertension. His blood pressure (BP) was typically high during these crises and often exceeded 200 mm Hg systolic BP. The episodes were associated with nausea, chest discomfort, epigastric pain, vomiting, sweating, headache, and occasional pharyngeal pain. The latter was described as a stabbing and severe pain located in the posterior part of the tongue, tonsillar fossa, and pharynx*.* The episodes started rather suddenly and lasted for 2–3 h, without any obvious precipitating factors, occurring with a frequency of one every 4–5 days. “Shaking” and prolonged fatigue following each episode were also described. A complete neurologic, neuropsychological, gastroenterological, cardiovascular, endocrinological, and nephrological workup did not reveal any abnormalities (Table 1). Measurements of metanephrines were repeated multiple times (Table 2). These values were either normal or slightly increased.

**Table 1:** Medical investigations performed to rule out the potential causes of hypertension, including the paroxysmal type

Disorders	Tests	Result
Adrenal-related		
Primary aldosteronism and mineralocorticoid excess syndromes	Serum aldosterone and renin, potassium	Unremarkable
Pheochromocytoma and paraganglioma	Serum total and free metanephrines, urinary metanephrines, serum and urinary catecholamines, abdominal MRI	Slight increase of 3-methoxytyramine during crisis
Adrenal congenital hyperplasia and Cushing syndrome	17(OH)-P, DOC, 11-deoxycortisol, A4, testosterone, DHEA-S, ACTH, serum cortisol, and late night salivary cortisol (2 times)	Unremarkable
(Para)thyroid-related		
Hyperparathyroidsm	Serum iPTH, calcium and phosphorus, 25(OH)D	Unremarkable
Hyperhypothyroidism	TSH, T3 and free T4 hormones	Unremarkable
Pituitary-related		
Acromegaly	GH and IGF-1	Unremarkable
Cushing disease	Late night salivary cortisol (2 times)	Unremarkable
Kidney-related		
Parenchymal or urinary tract disease	CBC, fasting glucose, lipid profile, serum creatinine with eGFR, UACR, electrolytes (sodium, potassium, calcium, phosphorus), serum UA, urinalysis, SPE and UPE	Unremarkable
Renal artery stenosis	Renal artery duplex ultrasonography	Unremarkable
Vascular-related		
Coarctation of aorta and other cardiac diseases	Ankle-brachial index, cardiovascular auscultation/palpation, 12-lead ECG, transthoracic echocardiogram	Unremarkable
Vasculitis and collagen vascular diseases	CRP, ESR, ANA, ANCA, Complement C3 and C4, liver and kidney function tests	Unremarkable
Neurogenic-related		
CNS lesions (stroke, tumor, hemorrhage, trauma, compression of lateral medulla)	Brain CT and MRI	Vascular compression of the left rostral ventrolateral medulla
Seizures and migraine	Scalp EEG × 2 Empiric anti-epileptic drugs Neurology consultation	Unremarkable
CNS infection	Lumbar puncture and CSF culture + PCR assays	Unremarkable
Others		
Systemic infections	CBC, CRP, serological tests (HBV, HIV, EBV, CMV, Lyme, TB)	Unremarkable
Drugs and toxins	Treatment review	Unremarkable
Psychogenic (labile hypertension, panic disorder)	Psychiatrist consultation	Unremarkable
Gastrointestinal conditions	CT chest-abdomen-pelvis, MR enterography, abdomen ultrasonography, esophageal manometry, gastric emptying scintigraphy, 24-h urinary 5-HIAA	Unremarkable

*17(OH)-P*, 17-hydroxyprogesterone; *25(OH)D*, 25-hydroxyvitamin D; *5-HIAA*, 5-hydroxyindoleacetic acid; *A4*, androstenedione; *ACTH*, adreno corticotropic hormone; *ANA*, antinuclear antibody; *ANCA*, antineutrophil autoantibodies; *CBC*, complete blood count; *CRP*, C-reactive protein; *CSF*, cerebro-spinal fluid; *DHEA-S*, dehydroepiandrosterone sulfate; *DOC*, 11-deoxycorticosterone; *eGFR*, estimated glomerular filtration rate; *ESR*, erythrocyte sedimentation rate; *GH*, growth hormone; *IGF-1*, insulin-like growth factor-1; *iPTH*, plasma intact PTH; *SPE*, serum protein electrophoresis; *TSH*, thyroid-stimulating hormone; *UA*, uric acid; *UACR*, urine albumin-to-creatinine ratio; *UPE*, urine protein electrophoresis

**Table 2:**
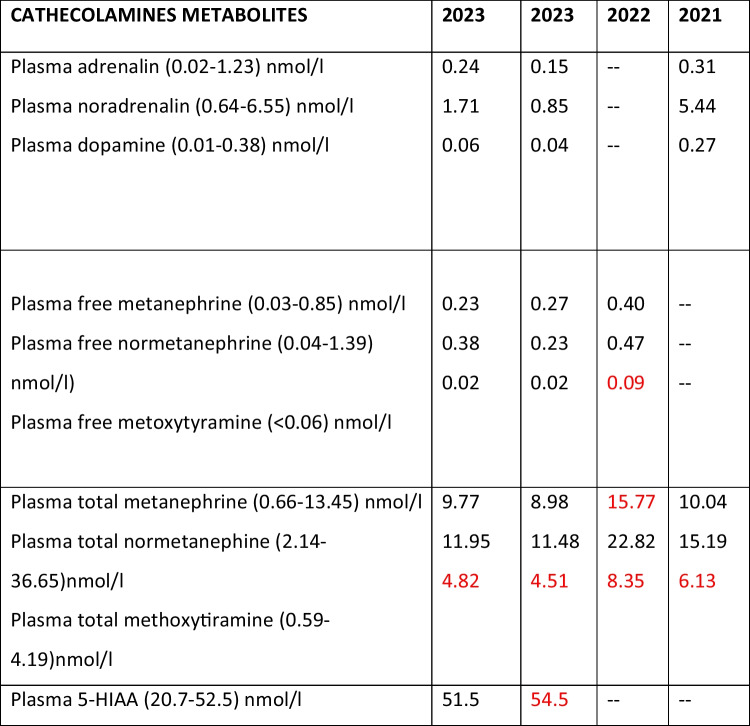
Preoperative measurements of catecholamines and their metabolites

5-HIAA, plasma 5-hydroxy-indoleacetic acid

Pathologic values are depicted in red, showing a slight elevation with respect to normal values. Last measurements, left column on the left, were obtained 6 months before the surgical procedure

**Fig. 1:**
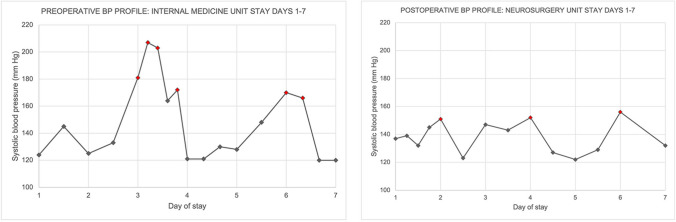
**A** Graphic showing the systolic blood pressure profile obtained during the preoperative stay in Internal Medicine Unit. Please note the episode of systolic BP exceeding the 200 mm Hg registered during a crisis. **B** Graphic showing the systolic BP profile obtained during the first post-operative week in Neurosurgery Unit. Please note the absence of hypertensive crisis

One of the episodes happened during hospitalization and the bedside examination revealed BP of 210/110 mm Hg not associated with flushing or pallor (Fig. 1A). The patient did not require any antihypertensive medication on a daily basis as he remained normotensive between the crises.

**Fig. 2:**
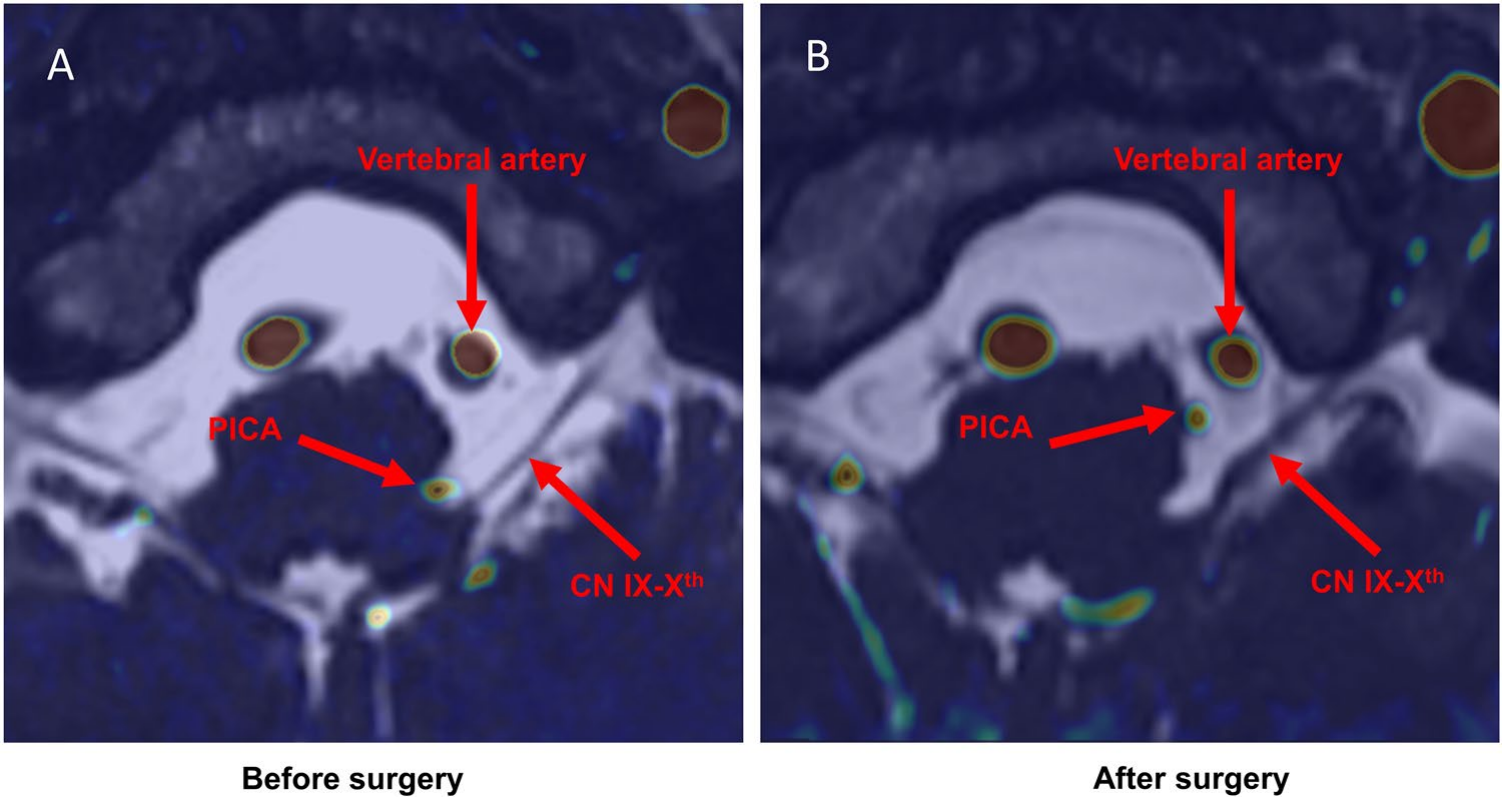
**A** Axial MRI, fusion between CISS and TOF sequences, showing the left PICA compressing the lateral aspect of the medulla oblongata. **B** Post-operative axial MRI, fusion between CISS and TOF sequences, showing that the PICA has been transposed with the lateral medulla being free

After 2 years of investigations, increasing frequency of symptomatic episodes (poorly controlled due to serious side effects associated to fast acting antihypertensive medications), a brain MRI was performed. The exam revealed a conflict between the left PICA and the RVLM at the level of the CN IX-X REZ (Fig. 2). A possible role of this neurovascular conflict was suggested, given the previous evidence [22]. We discussed with the patient the possibility to treat this rare syndrome with a MVD. Risks of the surgical procedure and uncertainties about the success rate were discussed. The patient consented to the proposed surgical procedure. The patient experienced a transient Xth nerve deficit (paralysis of left levator palatini muscle with consequent dysphagia) that resolved after 1 month. Values of BP, obtained during the post-operative phase, are represented in Fig. 1B. We obtained a reduction in terms of frequency (two episodes in the first post-operative month and one episode per month in the following period) and intensity of the episodes (systolic BP not exceeding 175 mm Hg) at 6-month follow-up (FU). The episodes were also described as significant less exhausting from the viewpoint of the patient that appeared globally satisfied with the proposed treatment.

## Description of the surgical technique

**Fig. 3:**
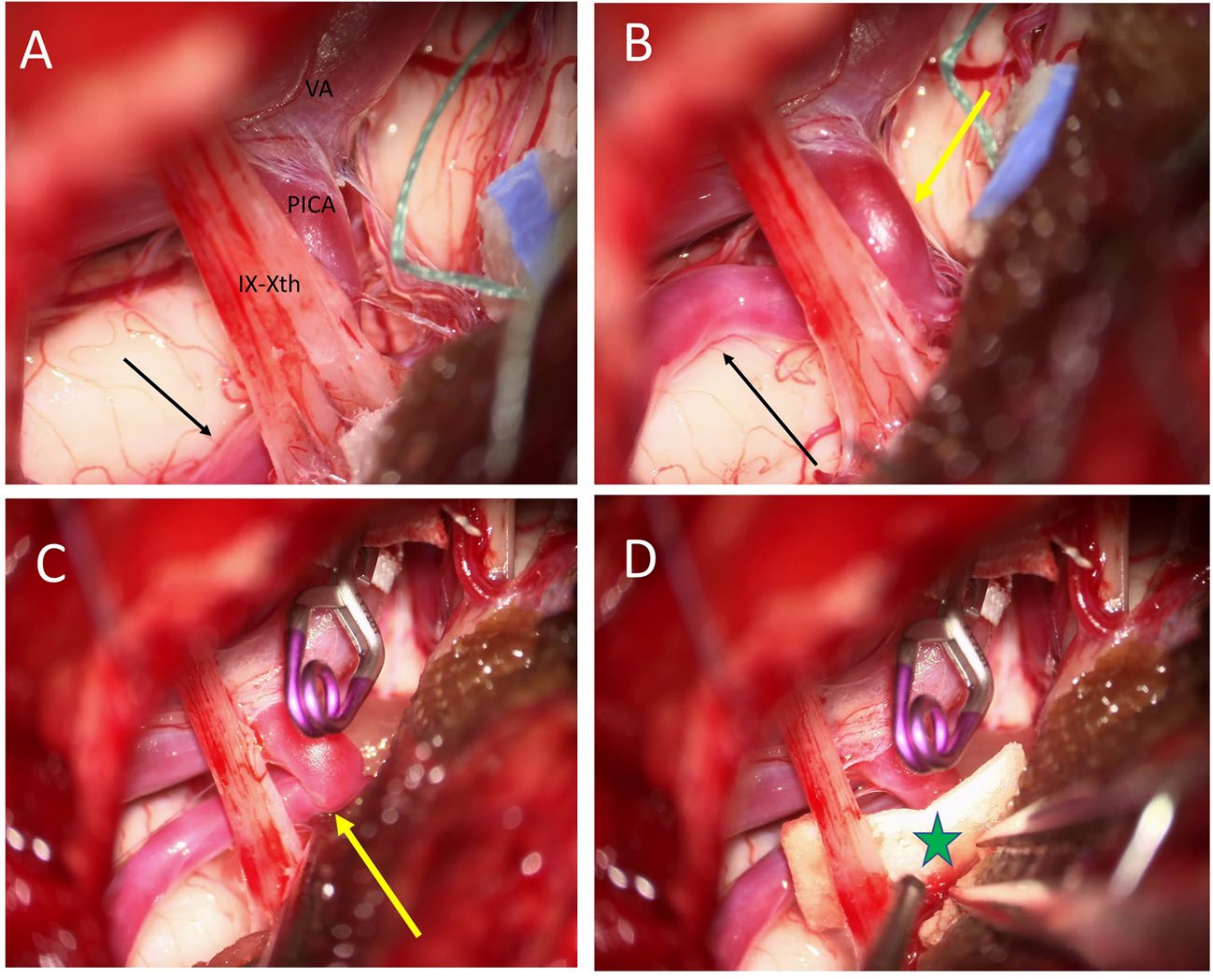
**A** Intraoperative image showing compression of the lateral medulla oblongata by the distal loop pf the PICA (black arrow). **B** The distal loop of the PICA has been dissected and moved away (black arrow) but there is still compression due to a proximal loop (yellow arrow). **C** A sling is wrapped around the vertebral artery and then secured with an aneurysm clip onto the petrous bone dura. This enables to relief the compression exerted by the proximal loop of the PICA (yellow arrow). **D** Teflon (green star) is positioned between the PICA and the medullary surface close to the root entry zone of lower cranial nerves

**Fig. 4:**
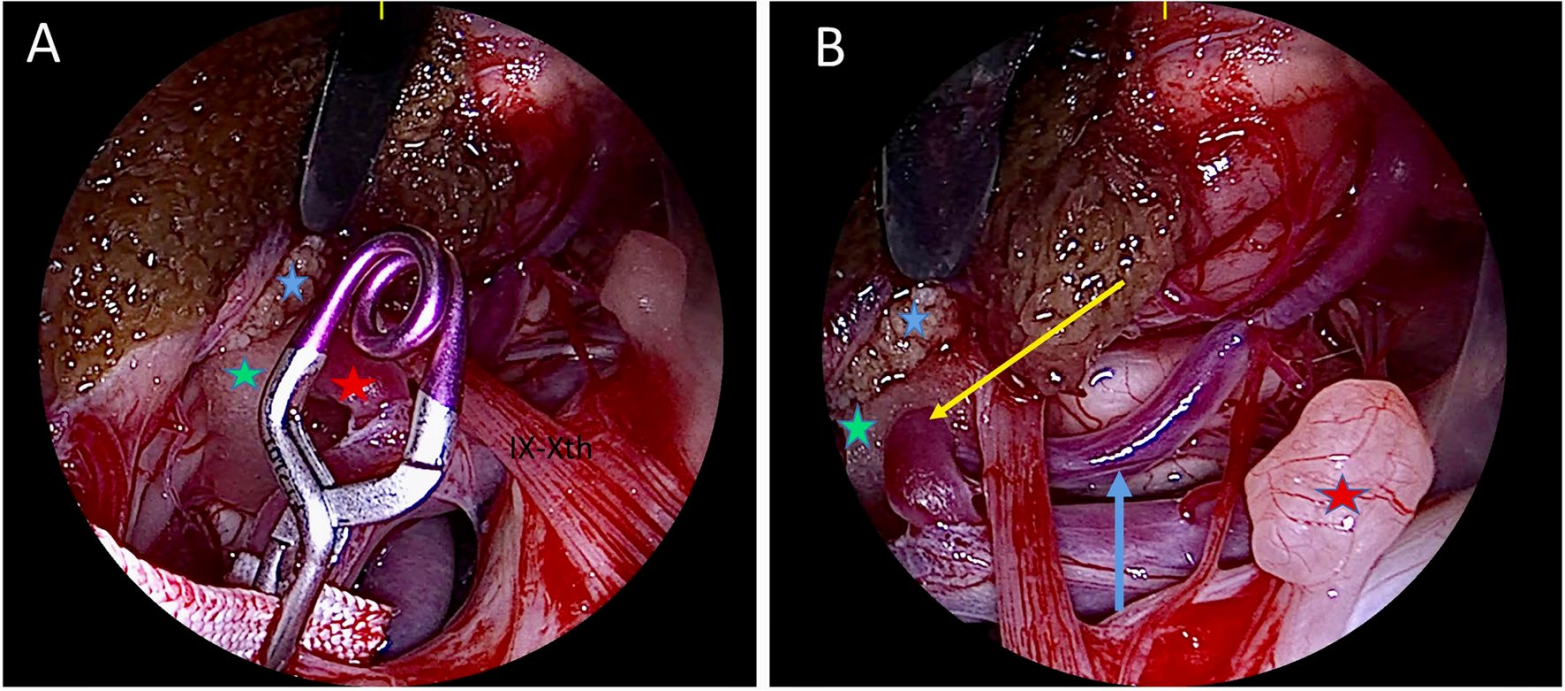
**A** Endoscopic view with 30° optic lens, observing from a lateral to medial perspective. The choroid plexus (blue star), the proximal loop of the PICA (red star), and the Teflon (green star) are visible. **B** The endoscope has been moved inferiorly. The choroid plexus (blue star) and the Teflon (green star) are identified. The proximal loop of the PICA (yellow arrow) and the distal loop (blue arrow) have been moved away from the brainstem

The patient was placed in lateral position with the head bent slightly toward the floor and then flexed avoiding jugular compression. Intraoperative neuromonitoring was used. A lazy-S skin retroauricolar incision was created. The superficial muscular layers were divided. A fat pad separating the superficial and deep muscular layers was encountered. The vertebral artery (VA, V3 segment) was identified. Transposition of the VA was not needed. A suboccipital craniotomy was performed. Extensive drilling of the supracondylar fossa was performed. The dura mater was opened. Extensive arachnoid opening was performed. The conflict was evident, between a loop of the PICA and the lateral medulla. The offending vessel was gently dissected away taking care of small perforators. A sling was wrapped around the VA and then secured with an aneurysm clip onto the petrous bone dura. Teflon was positioned between the PICA and the medullary surface close to the root entry zone of the IX–X cranial nerves (Fig. 3). The endoscope was used to check the absence of any residual conflict (Fig. 4). Video 1 illustrates the surgical procedure.

## Discussion

The RVLM contains the bulbospinal sympatho-excitatory neurons that play an important role in blood pressure and cardiovascular activity regulation [19]. Geiger et al. [8] published their experience with eight patients affected by refractory arterial hypertension that were treated by MVD. All of these patients experienced life-threatening hypertensive crises and were preoperatively taking three or more anti-hypertensive agents without control of BP or with intolerable side effects or both. The authors claimed a post-operative efficiency rate of 87.5%. Levy et al. [12] also described an 83% reduction in BP in a cohort of 12 patients. Frank et al. [7] later confirmed the effectiveness of MVD in patients affected by refractory hypertension but have demonstrated that the effect is transient. They postulated that this could be due to the long history of severe hypertension that can cause secondary end-organ damage and thus perturbs the regulation of neurohumoral and neural mechanisms. From 2010 forward, only a few reports have been published describing MVD for refractory hypertension mostly including case reports and very small surgical series [1, 20]. Sindou et al. described a prospective series of patients presenting hemifacial spasm associated with essential hypertension with an average FU of 7 years. They obtained BP normalization in the 58.33% of patients. Notably, they described a positive effect of MVD in significantly decreasing BP instability (from to 37.50 to 16.67% after surgery) and underlined that a positive effect could also be obtained in a delayed fashion in a certain amount of these patients [22]. Though the limited published clinical experience suggests a possible role for MVD in cases of refractory hypertension, many uncertainties remain [3]. This is related to the small sample size, low levels of evidence of the studies that mostly include case reports or retrospective case series, and with a short FU. To this latter point, studies with a longer FU seem to suggest a transient effect of MVD with the existence of a rebound phenomenon and return of BP to pre-surgical levels. The common denominator of all the previous published clinical experiences is that all patients presented with refractory hypertension with a pathologic baseline and eventually some hypertensive crises.

The clinical presentation including pharyngeal pain and digestive symptoms such as nausea, epigastric pain, and vomiting as well as the intraoperative findings of clear compression of CNs IX-X REZ (surgical video) allows to include the case in the frame of the vago-glossopharyngeal neuralgias though pharyngeal pain was not constantly present during each hypertensive crisis and was not described as the most prominent symptom by the patient [5]*.* The true peculiarity of the patient presented here is represented by the normal BP baseline (that does not allow us to refer to his condition as “refractory hypertension”) interspersed with episodic crises of severe hypertension lasting for several hours. This clearly distinguishes our case from the previous description found in the literature where patients presented an “essential hypertension” that was ultimately considered of neurogenic origin due to neurovascular compression and treated through MVD. Moreover, paroxysmal neurogenic hypertension has most commonly been described in association with diseases that diffusely affect the brainstem, such as tetanus, poliomyelitis, syringobulbia, and rarely to brainstem strokes [16, 18]. This suggests that this patient may be the first reported case of paroxysmal hypertension due to a neurovascular conflict, possibly strengthening the arguments in favor of a cause-effect relation between neurovascular conflicts and hypertension. Although an evident neurovascular conflict was diagnosed, many uncertainties persist, and we advise extreme caution with the interpretation of the MRI findings. Despite some studies reporting a statistically significant association between hypertension and a neurovascular conflict with the lateral medulla [2, 6, 17], more recent studies did not confirm this association [23]. A meta-analysis performed by Boogarts et al. [4] found that a neurovascular conflict was more prevalent in patients with apparent primary hypertension. However, considered individually, half of the studies did not find an association, and those that did were retrospective, unblinded, and with a small sample size. Moreover, no significant association was found when the subanalysis was limited to prospective studies [15]. Even though well-documented reports describing excellent results achieved with MVD [1, 9], the available literature does not enable to reach a definitive conclusion about the association between hypertension and vascular compression of the lateral medulla. Moreover, there are no unified criteria for screening and diagnosis of neurogenic hypertension and MRI interpretation is prone to subjectivity given the lack of standardized criteria to assess the compression of the lateral medulla. Considering our experience, we suggest to add a brain MRI with 3D CISS and TOF sequences [13] to the investigations usually performed in case of paroxysmal hypertension. If a clear conflict is identified, the possibility of performing MVD should be discussed with the patient. The risk of failure and the possibility of having symptom recurrence should be extensively discussed. The risks of surgery should also be discussed including facial palsy, deafness, lower cranial nerves injury, CSF leak, hemorrhage, stroke, and infection. Future studies are mandatory to obtain more robust conclusions about the association between neurogenic hypertension and a neurovascular conflict, possibly enabling a path forward to establish evidence-based criteria for safe and efficacious patients’ selection.

## Limitations

The literature concerning this subject lacks of robust evidence and criteria for patients’ selection are missing. Brainstem surgical manipulation and eventual RVLM-associated edema may result in a temporary effect on hypertension that wears off in time. The follow-up of the case here presented is very short and our results should be interpreted with caution.

## Conclusion

Brain MRI should be included in the diagnostic panel in cases of paroxysmal hypertension in order to exclude a neurovascular conflict. MVD can be considered in very selected patients presenting with intractable paroxysmal hypertension affecting quality of life when they exhibit a clear neurovascular conflict at the ventrolateral medulla. Limits of the surgical procedure need to be extensively discussed with the patient.

## Supplementary Information

Below is the link to the electronic supplementary material.

Supplementary file1 (MP4 144187 KB)

## Data Availability

Data are avalaible for consultation upon reasonable request.

## References

[1] Akaishi T, Kiyomoto H, Abe M, Okuda H, Ishizawa K, Endo T et al (2019) A 29-year-old woman with recurrent pregnancy-induced hypertension based on vascular compression of the medulla oblongata. Intern Med 58:2257–2261. 10.2169/internalmedicine.2382-18 30996172 10.2169/internalmedicine.2382-18 PMC6709317

[2] Akimura T, Furutani Y, Jimi Y, Saito K, Kashiwagi S, Kato S et al (1995) Essential hypertension and neurovascular compression at the ventrolateral medulla oblongata: MR evaluation. AJNR Am J Neuroradiol 16:401–405 7726090 PMC8338326

[3] Barley J, Ellis C (2013) Microvascular decompression: a surgical option for refractory hypertension of neurogenic etiology. Expert Rev Cardiovasc Ther 11:629–634. 10.1586/erc.13.30 23621144 10.1586/erc.13.30

[4] Boogaarts HD, Menovsky T, de Vries J, Verbeek ALM, Lenders JW, Grotenhuis JA (2012) Primary hypertension and neurovascular compression: a meta-analysis of magnetic resonance imaging studies. J Neurosurg 116:147–156. 10.3171/2011.7.JNS101378 21923244 10.3171/2011.7.JNS101378

[5] Chen J, Sindou M (2015) Vago-glossopharyngeal neuralgia: a literature review of neurosurgical experience. Acta Neurochir 157:311–321. 10.1007/s00701-014-2302-7 25526720 10.1007/s00701-014-2302-7

[6] Colón GP, Quint DJ, Dickinson LD, Brunberg JA, Jamerson KA, Hoff JT et al (1998) Magnetic resonance evaluation of ventrolateral medullary compression in essential hypertension. J Neurosurg 88:226–231. 10.3171/jns.1998.88.2.0226 9452228 10.3171/jns.1998.88.2.0226

[7] Frank H, Heusser K, Geiger H, Fahlbusch R, Naraghi R, Schobel HP (2009) Temporary reduction of blood pressure and sympathetic nerve activity in hypertensive patients after microvascular decompression. Stroke 40:47–51. 10.1161/STROKEAHA.108.518670 18927455 10.1161/STROKEAHA.108.518670

[8] Geiger H, Naraghi R, Schobel HP, Frank H, Sterzel RB, Fahlbusch R (1998) Decrease of blood pressure by ventrolateral medullary decompression in essential hypertension. Lancet 352:446–449. 10.1016/s0140-6736(97)11343-5 9708753 10.1016/s0140-6736(97)11343-5

[9] Hänggi D, Steiger H-J (2009) Symptomatic vertebral artery conflicts to the medulla oblongata and microsurgical treatment options: review of the literature. Neurosurg Rev 32:143–8. 10.1007/s10143-008-0182-0. (discussion 148-149) 19152014 10.1007/s10143-008-0182-0

[10] Jannetta PJ, Gendell HM (1979) Clinical observations on etiology of essential hypertension. Surg Forum 30:431–432 538657

[11] Kalia M, Fuxe K, Goldstein M (1985) Rat medulla oblongata. II. Dopaminergic, noradrenergic (A1 and A2) and adrenergic neurons, nerve fibers, and presumptive terminal processes. J Comp Neurol 233:308–32. 10.1002/cne.902330303 2858497 10.1002/cne.902330303

[12] Levy EI, Clyde B, McLaughlin MR, Jannetta PJ (1998) Microvascular decompression of the left lateral medulla oblongata for severe refractory neurogenic hypertension. Neurosurgery 43:1–6. 10.1097/00006123-199807000-00001. (discussion 6-9) 9657182 10.1097/00006123-199807000-00001

[13] Manava P, Naraghi R, Schmieder R, Fahlbusch R, Doerfler A, Lell MM et al (2021) 3D-visualization of neurovascular compression at the ventrolateral medulla in patients with arterial hypertension. Clin Neuroradiol 31:335–345. 10.1007/s00062-020-00916-z 32462236 10.1007/s00062-020-00916-z PMC8211615

[14] Mann SJ (2018) Neurogenic hypertension: pathophysiology, diagnosis and management. Clin Auton Res 28:363–374. 10.1007/s10286-018-0541-z 29974290 10.1007/s10286-018-0541-z

[15] Miller JP, Selman WR (2012) Hypertension and neurovascular compression. J Neurosurg 116:145–6. 10.3171/2011.4.JNS11549. (discussion 146) 21923242 10.3171/2011.4.JNS11549

[16] Montgomery BM (1961) The basilar artery hypertensive syndrome. Arch Intern Med 108:559–569. 10.1001/archinte.1961.03620100051007 14475539 10.1001/archinte.1961.03620100051007

[17] Naraghi R, Gaab MR, Walter GF, Kleineberg B (1992) Arterial hypertension and neurovascular compression at the ventrolateral medulla. A comparative microanatomical and pathological study. J Neurosurg 77:103–12. 10.3171/jns.1992.77.1.0103 1307855 10.3171/jns.1992.77.1.0103

[18] Phillips AM, Jardine DL, Parkin PJ, Hughes T, Ikram H (2000) Brain stem stroke causing baroreflex failure and paroxysmal hypertension. Stroke 31:1997–2001. 10.1161/01.str.31.8.1997 10926969 10.1161/01.str.31.8.1997

[19] Ruggiero DA, Cravo SL, Arango V, Reis DJ (1989) Central control of the circulation by the rostral ventrolateral reticular nucleus: anatomical substrates. Prog Brain Res 81:49–79. 10.1016/s0079-6123(08)61999-8 2694224 10.1016/s0079-6123(08)61999-8

[20] Sasaki S, Tanda S, Hatta T, Morimoto S, Takeda K, Kizu O et al (2011) Neurovascular decompression of the rostral ventrolateral medulla decreases blood pressure and sympathetic nerve activity in patients with refractory hypertension. J Clin Hypertens (Greenwich) 13:818–820. 10.1111/j.1751-7176.2011.00522.x 22051426 10.1111/j.1751-7176.2011.00522.x PMC8108941

[21] Sindou M (2015) Is there a place for microsurgical vascular decompression of the brainstem for apparent essential blood hypertension? A review Adv Tech Stand Neurosurg 42:69–76. 10.1007/978-3-319-09066-5_4 25411145 10.1007/978-3-319-09066-5_4

[22] Sindou M, Mahmoudi M, Brînzeu A (2015) Hypertension of neurogenic origin: effect of microvascular decompression of the CN IX-X root entry/exit zone and ventrolateral medulla on blood pressure in a prospective series of 48 patients with hemifacial spasm associated with essential hypertension. J Neurosurg 123:1405–1413. 10.3171/2014.12.JNS141775 26230479 10.3171/2014.12.JNS141775

[23] Zizka J, Ceral J, Elias P, Tintera J, Klzo L, Solar M et al (2004) Vascular compression of rostral medulla oblongata: prospective MR imaging study in hypertensive and normotensive subjects. Radiology 230:65–69. 10.1148/radiol.2301021285 14631051 10.1148/radiol.2301021285

